# Soluble Siglec-9 Improves Intestinal Barrier Function in a Mouse Model of Metabolic Dysfunction-Associated Steatohepatitis

**DOI:** 10.3390/metabo15060366

**Published:** 2025-05-30

**Authors:** Hisanori Muto, Fumitaka Mizuno, Takashi Honda, Shinya Yokoyama, Taku Tanaka, Kenta Yamamoto, Takanori Ito, Norihiro Imai, Yoji Ishizu, Kiyoshi Sakai, Hideharu Hibi, Masatoshi Ishigami, Hiroki Kawashima

**Affiliations:** 1 Department of Gastroenterology and Hepatology, Nagoya University Graduate School of Medicine, Nagoya 466-8550, Japan; hisanori.muto@fujita-hu.ac.jp (H.M.); mizuno.fumitaka.f7@f.mail.nagoya-u.ac.jp (F.M.); yokoyama.shinya.w3@f.mail.nagoya-u.ac.jp (S.Y.); t-tanaka@med.nagoya-u.ac.jp (T.T.);kenta-y@med.nagoya-u.ac.jp (K.Y.); ito.takanori.d9@f.mail.nagoya-u.ac.jp (T.I.); imai.norihiro.h8@f.mail.nagoya-u.ac.jp (N.I.); ishizu.yoji.n2@f.mail.nagoya-u.ac.jp (Y.I.); masaishi67115@gmail.com (M.I.); kawashima.hiroki.p5@f.mail.nagoya-u.ac.jp (H.K.); 2 Department of Gastroenterology and Hepatology, Fujita Health University Bantane Hospital, Nagoya 454-8509, Japan; 3 Department of Oral and Maxillofacial Surgery, Nagoya University Graduate School of Medicine, Nagoya 466-8550, Japan; www-kiyo@med.nagoya-u.ac.jp (K.S.); hibihi@med.nagoya-u.ac.jp (H.H.)

**Keywords:** chronic liver disease, gut–liver axis, hepatic steatosis, intestinal permeability, liver inflammation, MASH, metabolic dysfunction-associated steatotic liver disease, MASLD, soluble Siglec-9

## Abstract

**Background/Objectives:** Metabolic dysfunction-associated steatohepatitis (MASH), characterized by liver inflammation, fibrosis, and fat accumulation, can develop into cirrhosis and liver cancer. Despite its increasing prevalence worldwide, there are few established therapies for advanced MASH. We previously demonstrated that stem cells from human exfoliated deciduous teeth-conditioned media (SHED-CM) exerted therapeutic effects in a MASH mouse model. The gut–liver axis is thought to be associated with liver disease progression, and soluble Siglec-9 (sSiglec-9), an immunoinhibitory receptor, is a key protein in SHED-CM that induces anti-inflammatory macrophages and has intestinal epithelial protective effects. Therefore, we evaluated sSiglec-9’s role in intestinal barrier protection in MASH mice. **Methods**: We evaluated sSiglec-9 effects on intestinal barrier function using in vitro Caco-2 cell monolayers injured by TNF-α and IFN-γ. For the MASH mouse model, male C57BL/6J mice were given a Western diet and high-sugar solution orally; to induce liver injury, CCl4 was intraperitoneally administered for 12 weeks. Mice were treated weekly with 10 ng/g sSiglec-9 or vehicle. Intestinal permeability was assessed by blood 4 kDa FITC-dextran concentration, and intestinal transcriptomes and liver histology were analyzed. **Results**: sSiglec-9 decreased intestinal permeability and liver inflammation in MASH mice. sSiglec-9 and SHED-CM reduced 4 kDa FITC-dextran permeability in injured Caco-2 cells, and sSiglec-9 significantly reduced intestinal permeability and modulated expression of 34 intestinal genes. The NAFLD Activity Score indicated significantly reduced inflammation following sSiglec-9 treatment. **Conclusions**: sSiglec-9 may protect intestinal barrier function by mitigating mucosal inflammation. sSiglec-9 treatment may represent a novel therapeutic approach for MASH via gut–liver axis modulation.

## 1. Introduction

Metabolic dysfunction-associated steatotic liver disease (MASLD) encompasses hepatic phenotypes of metabolic syndrome [1,2,3,4]. Previously referred to as non-alcoholic fatty liver disease (NAFLD), MASLD is the leading cause of chronic liver disease worldwide and is characterized by the accumulation of fat in the liver (hepatic steatosis). Its prevalence has been increasing rapidly in recent years [1,2,3,4], often in parallel with the prevalence of obesity and obesity-related diseases such as type II diabetes [5]. Currently, MASLD affects approximately 25% of the general population, and about 25% of these cases progress to metabolic dysfunction-associated steatohepatitis (MASH), which is characterized by inflammation and fibrosis and has the potential to further advance to cirrhosis and liver cancer [6]. However, there are few established and effective pharmacological therapies for advanced MASH [7], and the development of new treatments remains a critical challenge [8].

In addition to fat accumulation in the liver, it is known that in MASLD/MASH, the interaction with extrahepatic organs such as adipose tissue and the intestinal tract is intricately related to the progression of the disease (multiple parallel hit theory) [9]. In recent years, the gut–liver axis has garnered increasing attention as a key pathway linking intestinal dysbiosis to liver disease progression [10]. Disruptions in gut microbiota homeostasis lead to increased intestinal permeability, allowing bacterial-derived molecules such as endotoxins to translocate into the portal circulation. These endotoxins subsequently stimulate hepatic macrophages, triggering the production of inflammatory mediators that induce chronic hepatocellular damage and activate hepatic stellate cells, which play a central role in fibrosis, ultimately leading to cirrhosis [11].

In a promising preclinical study of MASH, we have previously reported that injection of serum-free conditioned medium from stem cells derived from human exfoliated deciduous teeth (SHED-CM) provides therapeutic benefit in a MASH mouse model. This therapeutic benefit is multifaceted, including hepatoprotective, macrophage-mediated anti-inflammatory, and intestinal barrier-protective effects [12]. Among the factors contained in SHED-CM, previous studies have shown that soluble Siglec-9 (sSiglec-9) is an important factor that regulates macrophage polarization [13], and it has been reported that the survival rate of rats with D-galactosamine-induced acute hepatic failure is improved by administering Siglec-9 and MCP-1 [14]. Furthermore, in a mouse model of colitis, administration of soluble Siglec-9 reduces inflammation by inhibiting the NF-κB pathway [15].

Sialic acid-binding immunoglobulin-like lectins (Siglecs) are a family of receptors that are mainly expressed by immune cells, and they may promote cell–cell interactions and regulate immune cell function. Upon binding to terminal sialic acid residues on glycans, many Siglecs can initiate intracellular signaling [16,17,18]. Siglec-9 is an immunoinhibitory receptor that is mainly expressed on the cell surface of immune cells. Siglec-9 can bind to sialic acid ligands or agonistic antibodies, and it has shown potential as an immune checkpoint target for cancer immunotherapy [19,20]. Furthermore, sSiglec-9, an extracellular domain of Siglec-9, has been found to help reduce intestinal inflammation by inhibiting the NF-κB pathway [15]. Consequently, we propose that Siglec-9 may also provide utility as a therapeutic agent for MASH. In this study, we investigated the efficacy of sSiglec-9 in the MASH mouse model, focusing on its effect on intestinal barrier function.

## 2. Materials and Methods

### 2.1. Preparation of SHED-CM

Stem cells from human exfoliated deciduous teeth (SHEDs) were obtained following previously established protocols at Nagoya University School of Medicine [21], adhering to the institution’s approved guidelines (H-73, 2003). The cells were cultured in Dulbecco’s Modified Eagle’s Medium (DMEM) supplemented with 10% fetal bovine serum. When SHEDs reached 70–80% confluence at passages 8–12 (2.0 × 106 cells in a 10 cm dish), they were rinsed with phosphate-buffered saline (PBS), and the growth medium was substituted with serum-free DMEM. After a 48 h incubation period, the conditioned medium was harvested and subjected to centrifugation at 1500 rpm for 5 min. The resulting supernatant was collected and centrifuged again at 3000 rpm for 3 min. The final supernatant obtained from this process was designated as SHED-CM and utilized in subsequent experiments [12]. The ethical use of SHEDs was reviewed and approved by the ethics committee of Nagoya University, in compliance with the principles outlined in the Helsinki Declaration (2015–0278).

### 2.2. Caco-2 Monolayer Assay

Caco-2 cell experiments were performed as previously described [12]. Human Caco-2 cells were obtained from the American Type Culture Collection (Manassas, VA, USA). Caco-2 cells were cultured in DMEM supplemented with 10% fetal bovine serum. Cells were cultured in a humidified incubator at 5% CO_2_ and 37 °C. Cells were seeded at a density of 5 × 10^4^ cells/cm^2^ on a collagen-coated Transwell (Corning, New York, NY, USA) with a pore size of 0.4 μm and cultured as a monolayer before the experiment. The experiment was performed 14 days after seeding, when the cells had reached confluence and differentiation was complete. In the experiment, dysfunction was induced in differentiated Caco-2 monolayer cells by treatment with human recombinant IFN-γ (20 ng/mL, R&D Systems, Minneapolis, MN, USA) for 24 h, followed by application of human recombinant TNF-α (10 ng/mL, R&D Systems) to the basolateral compartment for 6 h. After inducing dysfunction in the cell monolayer, the medium in the basolateral chamber was replaced with serum-free DMEM containing 0, 100, or 400 ng/mL sSiglec-9 or SHED-CM, and the cells were cultured for 24 h. The internal control was expressed as cells cultured in DMEM without TNF-α or IFN-γ. The barrier function of the cell monolayers was determined by the apical to basolateral flux of 4 kDa fluorescein isothiocyanate (FITC)-dextran (Sigma-Aldrich, St. Louis, MO, USA). The apical chamber was filled with Hank’s balanced salt solution containing 1 mg/mL 4 kDa FITC-dextran, and the FITC-dextran concentration in the basolateral chamber was measured by spectrophotometer after 2 h.

### 2.3. Murine MASH Model and Treatment with sSiglec-9

To establish a mouse MASH model, we used 9-week-old male C57BL/6J mice obtained from Japan SLC (Shizuoka, Japan) and fed them a Western diet, which is closely associated with MASH in humans. This model was selected because gene expression and immune abnormalities were very similar to those of human MASH [22], and we followed previously reported protocols [23]. In short, these mice were fed a diet containing 21.1% fat, 41% sucrose, and 1.25% cholesterol (Teklad Diets; Envigo, Madison, WI, USA) and a high-concentration sugar solution (23.1 g/L d-fructose (Sigma-Aldrich, St. Louis, MO, USA) and 18.9 g/L d-glucose (Sigma-Aldrich, St. Louis, MO, USA). CCl4 (Wako, Osaka, Japan) was injected intraperitoneally once a week for 12 weeks at a dose of 0.2 μL (0.32 μg)/g body weight [23]. Before initiating the experiments, all mice were acclimated to the laboratory environment for two weeks. These mice also received 10 ng/g sSiglec-9 [15] or vehicle intraperitoneally once a week. The administration of sSiglec-9 or vehicle was performed 3 days after each CCl_4_ injection. The intestinal permeability assay was conducted at week 12, and all mice were euthanized 3 days later under deep anesthesia. Four animals were assigned to each treatment group (*n* = 4 per group). Liver and cecum were collected for histological and gene expression analysis. Cecal contents were also collected for microbiome analysis. The animal experimental protocol was approved by the Institutional Animal Care and Use Committee of Nagoya University (M220028-002). In addition, all experiments performed in this study conformed to the National Institutes of Health guidelines for the care and use of laboratory animals and the ARRIVE guidelines.

### 2.4. Intestinal Permeability

The intestinal permeability was evaluated using the methods previously described [24]. The mice were fasted for 4 h, and 4 kDa FITC-dextran (20 mg/mL, PBS) was administered orally at a dose of 10 μL/kgBW. After 4 h, blood from the retrobulbar capillary plexus was sampled into heparinized tubes for 4 kDa FITC-dextran analyses. Plasma was obtained after centrifugation at 2000× *g* for 5 min. Plasma was diluted 1:5 (*v*/*v*) in PBS. Fluorescence was measured spectrophotometrically (BioTek Cytation; Agilent, Tokyo, Japan) in 96-well plates (excitation: 485 nm, emission: 528 nm). FITC-dextran concentrations were calculated using standard concentrations prepared in PBS ranging from 0 to 2.5 µg/mL 4 kDa FITC-dextran. Emission signals in plasma were calculated by subtraction of those of mice treated with the 4 kDa FITC-dextran from those received by PBS.

### 2.5. Histological Analyses

Liver tissues fixed in formalin and embedded in paraffin were sliced into sections with a thickness of 4 μm, followed by staining with hematoxylin-eosin (H&E) and Sirius red. The tissues were observed and evaluated using a BZ-X800 microscope (Keyence, Osaka, Japan). The NAFLD Activity Score (NAS) was calculated by adding the individual scores assigned for steatosis (ranging from 0 to 3), hepatocellular ballooning (scored 0 to 2), and lobular inflammation (scored 0 to 3) [25].

### 2.6. RNA Sequencing Analysis

Total RNA was extracted from frozen liver or ileum tissues using the RNeasy Mini Kit (Qiagen, Venlo, The Netherlands) according to the manufacturer’s instructions. Extracted total RNA was quality controlled using the TapeStation (Agilent, Santa Clara, CA, USA).

Poly A selection using the NEBNext^®^ Poly(A) mRNA Magnetic Isolation Module (New England Biolabs, Ipswich, MA, USA) and sequencing library preparation using the NEBNext^®^ UltraTM ll Directional RNA Library Prep Kit (New England Biolabs, Ipswich, MA, USA) and data acquisition using NovaSeq 6000 (Illumina, San Diego, CA, USA) were performed by Rhelixa (Tokyo, Japan). Primary analysis of the 8 RNA-seq raw data was performed using RaNa-seq [26] on the web, and raw count data were output. Secondary analysis of the RNA-seq data was performed on the web by inputting these count data into RNAseqChef [27]. For pairwise comparison analysis, differentially expressed genes (DEGs) detection was performed using edgeR with a false discovery rate (FDR) cutoff of 2 and less than 0.05, respectively. Functional enrichment analysis was performed based on the GO Biological Process gene set.

### 2.7. Analysis of Gut Microbiota

Fecal samples were obtained from the cecum on the day of sacrifice, frozen, and stored at −80 °C immediately. DNA was isolated using the DNeasy PowerSoil Kit (Qiagen, Hilden, Germany). Isolated DNA was amplified to target the V3–4 regions of bacterial 16S rRNA as previously described [28]. PCR products were pooled to construct the sequencing library, which was then sequenced using MiSeq (Illumina, San Diego, CA, USA). For basic analysis of the 16S rRNA gene sequence data, Quantitative Insights Into Microbial Ecology (QIIME 2–2024.5 with DADA2) [29] and SILVA (version 138) were used. Linear discriminant analysis effect size (LEfSe) [30] was used to compare microbiome compositions. The alpha diversity of the gut microbiota, which indicates species richness, was analyzed using the Chao1, Observed, and Shannon methods with MicrobiomeAnalyst [31].

### 2.8. Statistical Analysis

The GraphPad Prism version 9.2.0 (GraphPad Software, Boston, MA, USA) was used for statistical analyses. Data are presented as bar graphs showing mean ± standard error of the mean. The two groups were compared using Student’s *t* test for continuous variables. A *p*-value < 0.05 was considered significant.

## 3. Results

### 3.1. sSiglec-9 Restores Caco-2 Monolayer Dysfunction In Vitro

**Figure 1:**
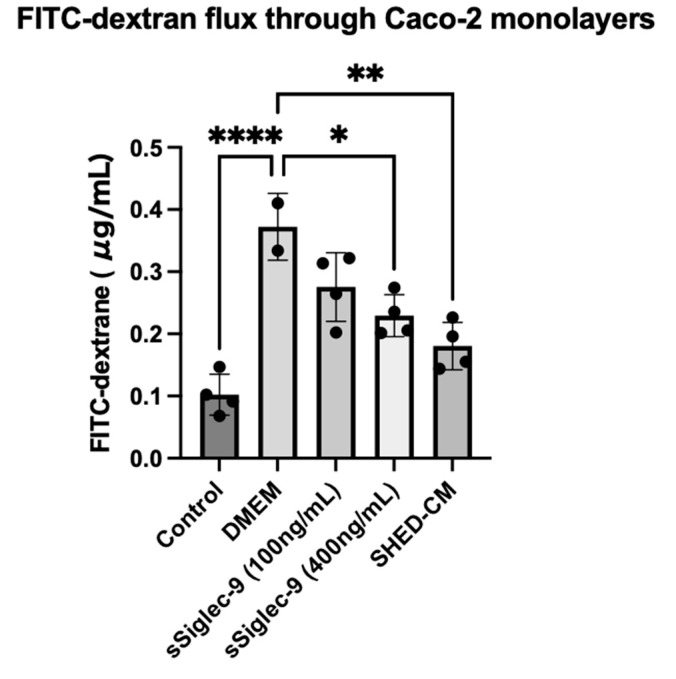
Quantification of paracellular FITC-dextran flux through Caco-2 monolayers treated with IFN-γ and TNF-α. The control group consists of cells cultured in DMEM without TNF-α or IFN-γ. Treatment with SHED-CM (*p* = 0.0012) and 400 ng/mL sSiglec-9 (*p* = 0.0126) significantly reduced Caco-2 monolayer damage. Statistical analysis was performed using one-way ANOVA followed by Tukey’s multiple comparisons test. Data are presented as mean ± SEM. * *p* < 0.05, ** *p* < 0.01, **** *p* < 0.0001.

To investigate the effect of Siglec-9 on intestinal barrier function, we used Caco-2 monolayer cells treated with IFN-γ and TNF-α as an in vitro model. The permeability of 4kDa FITC-dextran, a parameter of intercellular permeability of uncharged macromolecules, was significantly increased by treatment with IFN-γ and TNF-α (*p* < 0.0001). Treatment with SHED-CM significantly suppressed the increase in intercellular FITC-dextran flux caused by IFN-γ and TNF-α (*p* = 0.0012, Figure 1). Furthermore, treatment with sSiglec-9 at a concentration of 400 ng/mL was found to have a protective effect on the function of Caco-2 monolayer cells (*p* = 0.0126), similar to that of SHED-CM.

### 3.2. Establishment of the Murine MASH Model

**Figure 2:**
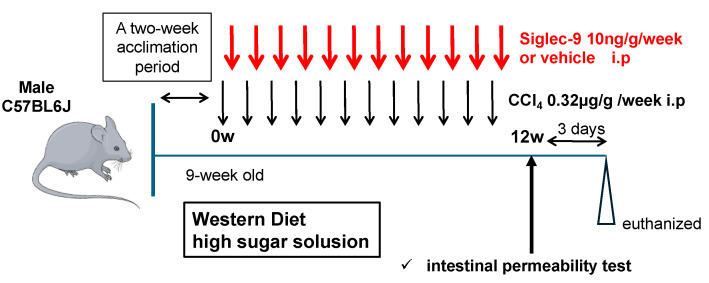
Protocol of the in vivo assay. Nine-week-old male C57BL/6J mice were fed a Western diet and a high-sugar solution. CCl_4_ was administered intraperitoneally once per week for 12 weeks. These mice also received intraperitoneal injections of 10 ng/g sSiglec-9 or PBS once per week. Intestinal permeability assessment was performed, and the mice were sacrificed two days later for blood analysis, gene expression profiling, histological examination, and gut microbiota analysis.

Figure 2 shows the animal experiment protocol used in this study. This mouse MASH model had an expression profile that was very similar to that of human MASH, and it was induced using the same method as that previously reported [23].

### 3.3. sSiglec-9 Protects the Intestinal Barrier in the MASH Mouse Model

**Figure 3:**
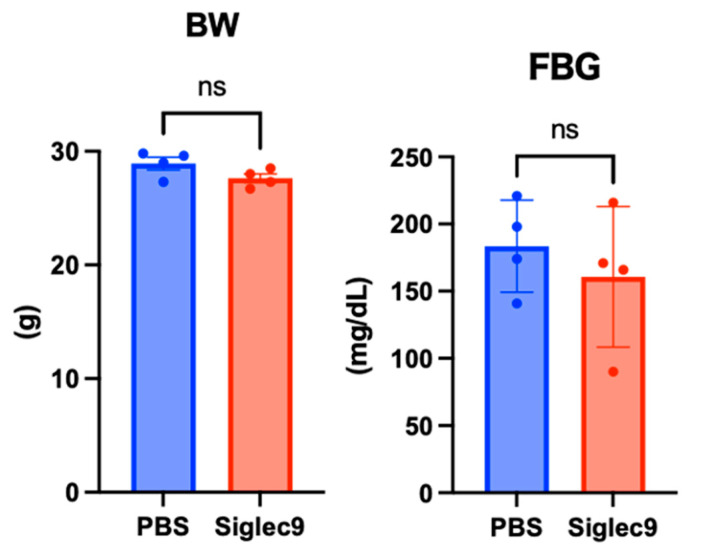
Body weight (BW) and fasting blood glucose (FBG) of mice at the time of intestinal permeability assessment. There were no significant differences in BW and FBG between the two groups (*p* = 0.109 and *p* = 0.494, respectively). ns: not significant (*p* > 0.05).

**Figure 4:**
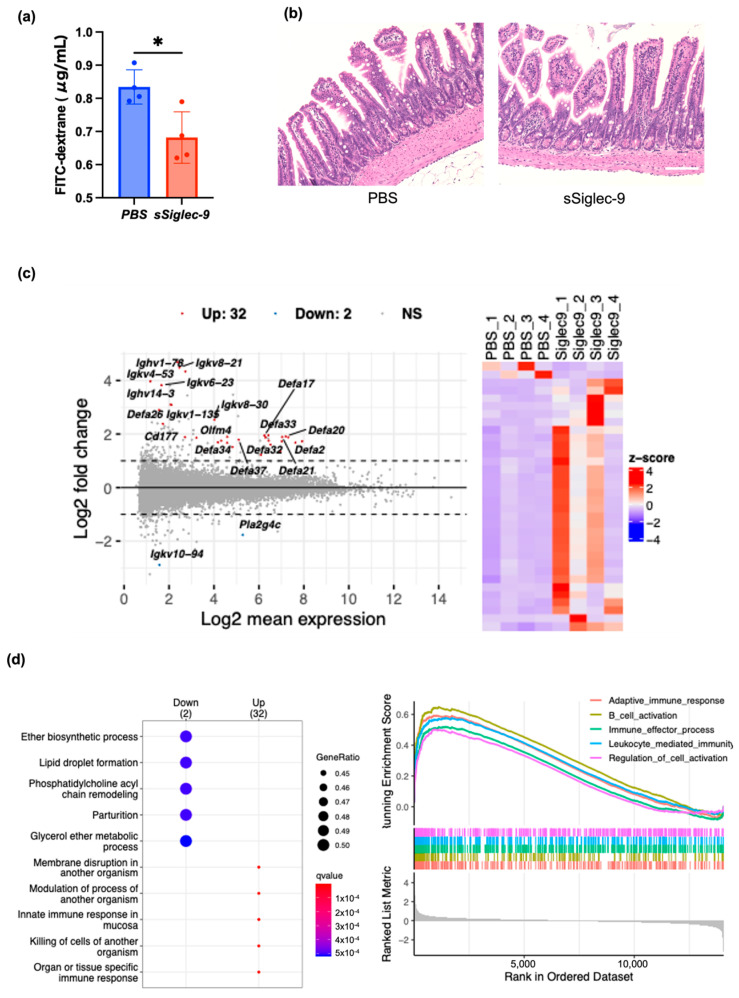
sSiglec-9 administration restores intestinal barrier function and alters gene expression in ileal tissue. sSiglec-9 treatment not only improves intestinal permeability but also induces significant transcriptional changes in the ileum, particularly in genes related to immune function. (**a**) Concentration of 4 kDa FITC-dextran in blood, reflecting intestinal barrier function, following oral administration to mice. Administration of sSiglec-9 significantly restored intestinal barrier function (*p* = 0.016). Data are presented as mean ± SEM. * *p* < 0.05. (**b**) Representative images of the macroscopic appearance of H&E-stained intestines. No obvious differences were observed between the two groups. Scale bar: 100 μm. (**c**) MA plot and heatmap illustrating differentially expressed genes (DEGs) from RNA sequencing analysis of ileal tissue. A total of 32 genes, including several α-defensin genes and Immunoglobulin kappa variable gene, were significantly upregulated following sSiglec-9 administration, while 2 genes were significantly downregulated (fold change > 2, false discovery rate < 0.05). (**d**) Results of enrichment analysis using the GO Biological Process gene set in ileal tissue. Over-representation analysis revealed an upregulation of pathways such as ‘membrane disruption in another organism’, ‘modulation of process of another organism’, and ‘innate immune response in mucosa’ in the sSiglec-9 group. Gene set enrichment analysis (GSEA) identified increased enrichment of pathways including ‘adaptive immune response’, ‘B cell activation’, and ‘leukocyte-mediated immunity’ following sSiglec-9 administration.

Next, we investigated the effect of sSiglec-9 on intestinal permeability in the mouse MASH model. The blood concentration of 4 kDa FITC-dextran after oral administration, which reflects intestinal permeability, exceeded the value observed in a single normal mouse housed under the same conditions (0.586 µg/mL) in all MASH model mice used in the experiment. This finding suggests the occurrence of intestinal barrier dysfunction in MASH. At 12 weeks, there were no apparent changes in the body weight or fasting blood glucose levels of the mice with or without sSiglec-9 administration (Figure 3). Intestinal permeability results showed that the intestinal barrier function was significantly protected by sSiglec-9 (*p* = 0.017, Figure 4a). H&E staining of ileal tissue revealed no substantial inflammatory cell infiltration in any of the groups, indicating normal tissue morphology. Additionally, no differences were observed in villus height or the number of Paneth cells between groups (Figure 4b). However, RNA sequencing analysis identified transcriptomic changes in intestinal tissue following sSiglec-9 administration [19]. In the sSiglec-9 group, 32 genes were upregulated and 2 genes were downregulated (fold change > 2, false discovery rate < 0.05, Figure 4c, Table S1). GO analysis showed that the 32 upregulated genes, including α-defensin genes and Immunoglobulin kappa variable genes, were related to intestinal mucosal immunity, such as “cell membrane disruption in other organisms” and “innate immune response in mucosa” (Figure 4d).

### 3.4. sSiglec-9 Attenuates Liver Inflammation in the MASH Mouse Model

**Figure 5:**
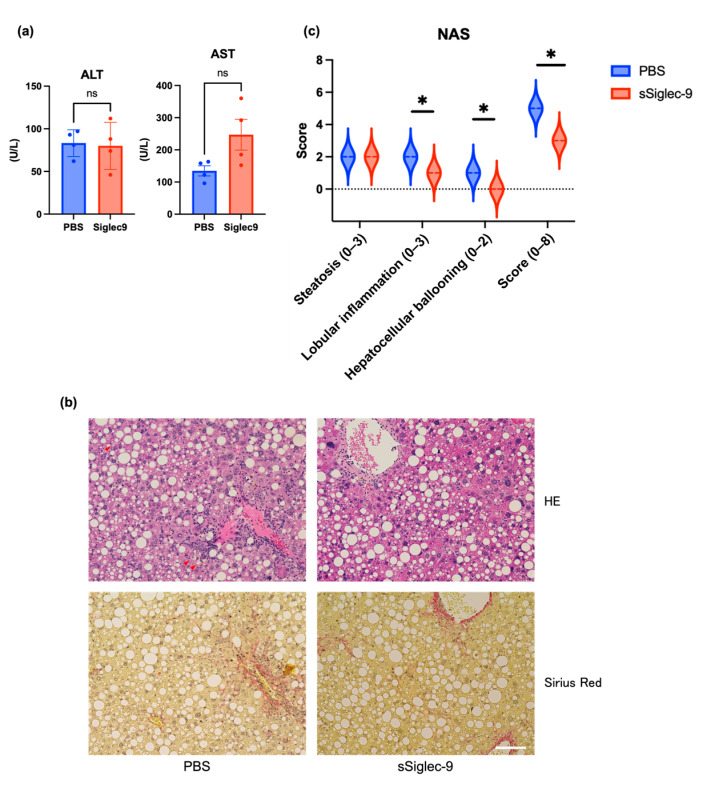
sSiglec-9 administration ameliorates liver inflammation and histological severity in MASH mice. While no significant changes were observed in serum ALT and AST levels, sSiglec-9 treatment improved histopathological markers of liver injury. (**a**) Serum alanine aminotransferase (ALT) and aspartate aminotransferase (AST) levels at the time of sacrifice. No significant differences were observed between the two groups (*p* = 0.845 and *p* = 0.068, respectively). Data are presented as mean ± SEM. (**b**) Representative images of the macroscopic appearance of H&E-stained and Sirius Red-stained livers. Arrowheads indicate hepatocellular ballooning. No obvious differences in the fibrosis area were observed between the two groups. Scale bar: 100 μm. (**c**) Comparison of NAFLD Activity Score (NAS). sSiglec-9 administration significantly reduced lobular inflammation, hepatocellular ballooning, and the total NAS (*p* = 0.029 each), as indicated by asterisks (*).

**Figure 6:**
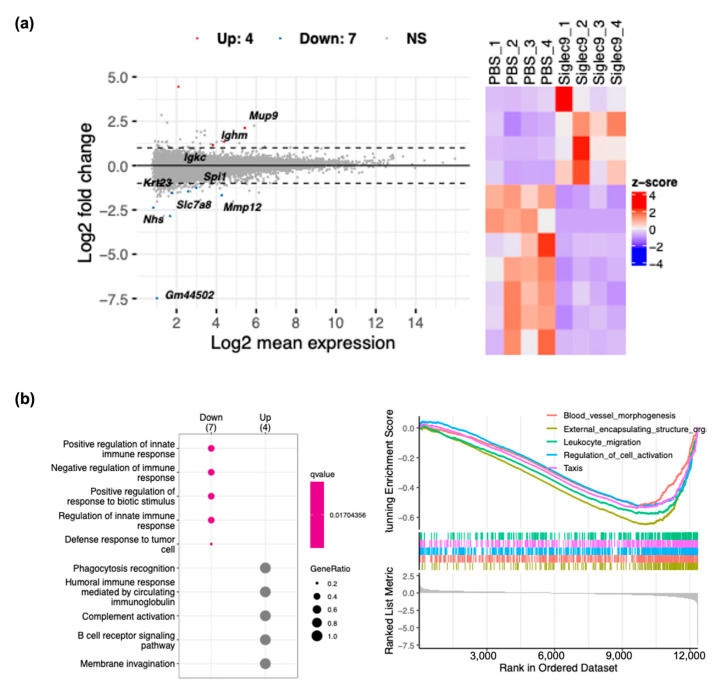
sSiglec-9 administration modulates liver gene expression and immune-related pathways. Transcriptomic analysis of liver tissue revealed distinct gene expression changes following sSiglec-9 treatment, suggesting an impact on immune regulation. (**a**) MA plot and heatmap illustrating differentially expressed genes (DEGs) from RNA sequencing analysis of liver tissue. Four genes were significantly upregulated following sSiglec-9 administration, while seven genes were significantly downregulated (fold change > 2, false discovery rate < 0.05). (**b**) Results of enrichment analysis using the GO Biological Process gene set in liver tissue. Over-representation analysis revealed a downregulation of pathways such as ‘positive regulation of innate immune response’, ‘negative regulation of immune response’, and ‘positive regulation of response to biotic stimulus’ in the sSiglec-9 group. Gene set enrichment analysis (GSEA) identified decreased enrichment of pathways including ‘blood vessel morphogenesis’, ‘external encapsulating structure organization’, and ‘leukocyte migration’ following sSiglec-9 administration.

The liver of vehicle-treated mice exhibited histological changes resembling human MASH, with a median steatosis score of 2, lobular inflammation of 2, ballooning of 2, and fibrosis stage of 2; this confirmed the successful establishment of the MASH mouse model. Regarding liver inflammation, sSiglec-9 administration did not result in significant changes in serum alanine aminotransferase (ALT) or aspartate aminotransferase (AST) levels (*p* = 0.845 and *p* = 0.068, respectively, Figure 5a). Similarly, no substantial improvement in liver fibrosis was observed based on histological assessment (Figure 5b). However, evaluation using the NAS indicated a significant reduction in inflammation following sSiglec-9 treatment (*p* = 0.029). No substantial changes were observed in hepatic steatosis (Figure 5b,c). Transcriptome analysis of the liver tissue identified 11 DEGs (fold change > 2, false discovery rate < 0.05, Figure 6a, Table S2). Enrichment analysis of the seven genes whose expression was decreased revealed a significant enrichment of genes related to innate immunity, such as “positive regulation of response to biotic stimulus” (Figure 6b).

### 3.5. sSiglec-9 Treatment Does Not Alter Diversity of the Gut Microbiota

**Figure 7:**
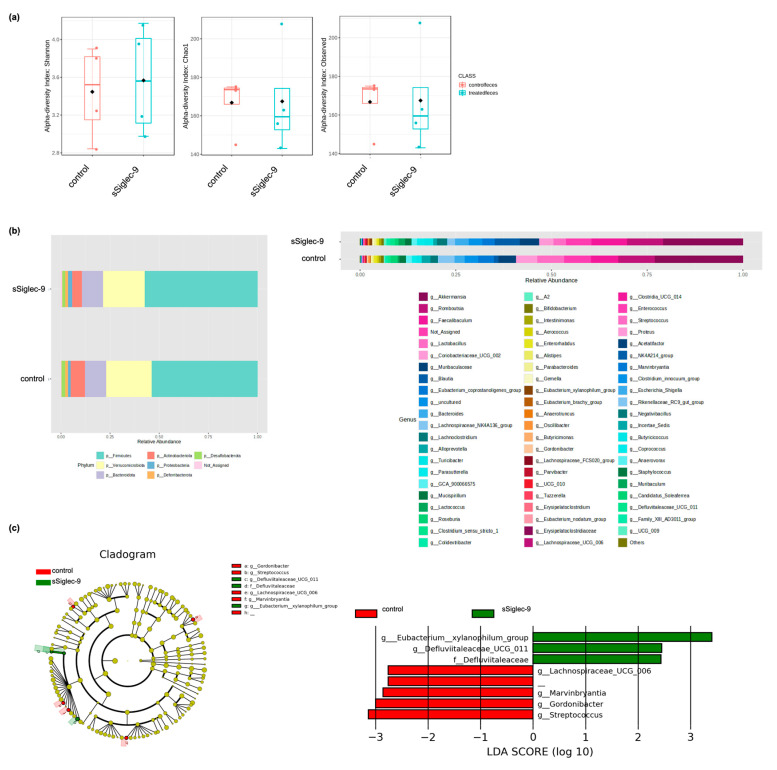
Effects of sSiglec-9 administration on cecal microbiota composition. Analysis of the cecal microbiota revealed no significant differences in α-diversity or overall taxonomic composition between the sSiglec-9 and control groups, while specific bacterial taxa exhibited enrichment or depletion following treatment. (**a**) Comparison of α-diversity in cecal microbiota analysis. No significant differences were observed between the two groups using Chao1, Shannon, or observed species indices (*p* > 0.99 each). (**b**) Microbiome proportions at the phylum and genus levels in the sSiglec-9 and control groups. No substantial differences in overall composition were observed between the two groups. (**c**) Linear Discriminant Analysis Effect Size (LEfSe) analysis results. In the sSiglec-9 treatment group, *g_Eubacterium_xylanophilum_group*, *g_Defluviitaleaceae_UCG_011*, and *f_Defluviitaleaceae* were enriched. Conversely, *g_Streptococcus*, *g_Gordonibacter*, *g_Marvinbryntia*, and *g_Lachnospiraceae UCG_006* were depleted.

There were no significant changes in the alpha diversity of the gut microbiota following Siglec-9 treatment (Figure 7a). The microbiome profiles of each group after treatment are shown in Figure 7b at both the phylum and genus levels, indicating no substantial differences in overall composition. However, Lefse analysis revealed that the sSiglec-9 group exhibited a higher abundance of bacteria belonging to the genus *Eubacterium xylanophilum*, the genus *Defluviitaleaceae UCG-011*, and the family *Defluviitaleaceae*. Additionally, the relative abundance of the genus *Streptococcus* was reduced in the sSiglec-9 group (Figure 7c).

## 4. Discussion

MASLD and MASH are rapidly increasing worldwide and, despite being a major public health concern, have limited effective drug treatments [32,33]. In this study, we demonstrated that sSiglec-9, a key bioactive factor present in SHED-CM, exerts protective effects on intestinal barrier function and attenuates liver inflammation in a MASH mouse model. Our findings suggest that sSiglec-9 may play a pivotal role in mediating the therapeutic benefits previously observed with SHED-CM, particularly through its influence on the gut–liver axis.

The intestinal barrier has critical roles as a physical barrier and is thought to represent an important line of defense against external injuries. Intestinal barrier dysfunction, such as from tight junction losses and antimicrobial peptide changes, can increase permeation of the intestine to pathogen-associated molecular patterns such as bacterial endotoxins and induce Toll-like receptor signaling cascades in the liver. These processes underlie the development of MASLD. Consequently, the involvement of intestinal barrier function in MASLD/MASH pathogenesis has become increasingly recognized. However, it remains unclear how the associated pathways can be targeted to treat MASH [34].

SHED-CM has shown promise in suppressing liver fibrosis in MASH models through anti-inflammatory and intestinal barrier-protective effects [12]. Among its numerous bioactive factors [13], sSiglec-9 is particularly abundant and has been implicated in regulating macrophage polarization and improving survival in rodent models of acute liver failure [14]. Given these functions, sSiglec-9 is likely a key contributor to the hepatoprotective effects of SHED-CM.

Siglecs are receptors expressed on innate immune cells that recognize sialic acid residues and regulate immune responses [16]. Their inhibitory signaling mechanisms suppress pro-inflammatory cytokines and promote anti-inflammatory responses [18]. The interaction between sialylated glycans and Siglecs plays a crucial role in maintaining immune homeostasis and controlling inflammation, particularly in the gut [17,35].

Siglec-9 has been reported to inhibit inflammation through immunoreceptor tyrosine-based inhibitory motifs and by contributing to the production of IL-10 in macrophages [36,37]. Additionally, sSiglec-9 has been shown to exert potent anti-inflammatory effects by suppressing NF-κB-mediated inflammatory responses in intestinal epithelial cells, reducing the production of pro-inflammatory cytokines such as IL-8 and TNF-α [15]. Consequently, sSiglec-9 may influence the intestinal barrier because of its impact on cytokines and immune cells. Moreover, it has been found to alleviate intestinal inflammation in mouse models of colitis [15,38], highlighting its potential as a therapeutic target for inflammation-related diseases, including MASH.

In this study, sSiglec-9 reversed the increased intestinal permeability in the MASH mouse model. At the same time, RNA sequencing analysis revealed an upregulation of multiple α-defensin genes and IgKV genes. Among these, α-defensin, an antimicrobial peptide secreted by Paneth cells, plays a crucial role in maintaining the intestinal microbiota homeostasis [38]. In the context of MASH research, a decrease in intestinal α-defensin has been reported to exacerbate liver fibrosis by promoting dysbiosis and disrupting the intestinal environment [39]. The upregulation of α-defensin by sSiglec-9 may therefore contribute to the amelioration of intestinal inflammation. This hypothesis aligns with previous findings indicating that reduced α-defensin expression is associated with ileal mucosal inflammation, such as in Crohn’s disease [40]. Additionally, the upregulation of IgKV genes may contribute to the strengthening of the intestinal barrier and the activation of adaptive immunity. B cells, primarily found in Peyer’s patches and mesenteric lymph nodes, produce IgA, which plays a crucial role in mucosal immunity by preventing the adhesion and invasion of intestinal bacteria and pathogens [41]. Siglec-9 is also known to induce anti-inflammatory macrophages that secrete IL-10 [14], a key mediator in intestinal homeostasis and B-cell regulation [42,43]. On the basis of these observations, sSiglec-9 emerges as a promising therapeutic target for moderating the “gut–liver axis”, which links the intestine and liver, and may represent a novel therapeutic strategy for MASH.

Regarding the gut microbiota analysis, there was no significant difference in alpha diversity between the sSiglec-9 treatment group and the control group. This suggests that the primary mechanism underlying sSiglen-9-mediated disease improvement may not be through the correction of dysbiosis. This finding is also consistent with previous studies on SHED-CM. However, Lefse analysis indicated an increased abundance of *Eubacterium xylanophilum* and *Defluviitaleaceae UCG-011* following sSiglec-9 treatment. Bacteria of the genus Eubacterium produce short-chain fatty acids (SCFAs), particularly butyrate, which play essential roles in energy metabolism, intestinal motility, immune regulation, and anti-inflammatory processes [44]. *Defluviitaleaceae UCG-011* has also been suggested to have potential health benefits [45,46,47]. Notably, the relative abundance of *Streptococcus* was lower in the sSiglec-9 group. This is particularly intriguing, as previous studies have reported an increased presence of *Streptococcus* in patients with advanced liver cirrhosis [48,49,50]. While these microbial changes may be relevant to the therapeutic effects of sSiglec-9, their precise role remains unclear and warrants further investigation.

In liver tissue, administration of sSiglec-9 alleviated inflammation, and RNA sequencing analysis confirmed the downregulation of a gene set related to innate immunity. While the underlying immunoregulatory mechanisms—such as potential effects on hepatic macrophages or cytokine production—remain to be elucidated, and it is uncertain whether these hepatic effects are directly mediated by sSiglec-9 or are secondary to improved intestinal barrier function, these findings nonetheless support the therapeutic mechanism of sSiglec-9 via modulation of the gut–liver axis. Despite these benefits, no substantial improvement in liver fibrosis was observed. These results emphasize that the primary therapeutic action of sSiglec-9 may lie in its ability to restore intestinal barrier integrity rather than to induce systemic immunomodulation. Given the increasing recognition of the intestinal barrier as a critical therapeutic target in MASH, the robust barrier-protective effects observed here highlight the translational potential of sSiglec-9 in future clinical applications.

In contrast, previous research demonstrated that SHED-CM significantly attenuated fibrosis in the MASH mouse model [12], suggesting that factors other than sSiglec-9 contribute to its antifibrotic effects. Notably, a study using a mouse model of carbon tetrachloride-induced liver fibrosis identified hepatocyte growth factor (HGF) as a key mediator of the antifibrotic properties of SHED-CM [51]. These findings highlight the need for further investigation into the specific roles of the various bioactive components present in SHED-CM. While the therapeutic effects of sSiglec-9 were modest compared with the broader benefits observed with SHED-CM, our findings underscore its potential as a novel therapeutic agent targeting both intestinal and hepatic pathology in MASH. Future studies should focus on elucidating the precise molecular mechanisms underlying the effects of sSiglec-9, optimizing dosing strategies, and exploring its efficacy in combination with other therapeutic agents.

This study has several methodological limitations. First, we assessed intestinal barrier function using gene expression analysis and FITC-dextran assays; these methods may not fully capture the complexity of barrier dynamics. In addition, the underlying mechanisms by which sSiglec-9 exerts its therapeutic effects remain insufficiently explored. For example, we did not validate the RNA-seq findings at the protein level using immunohistochemistry or Western blot, particularly for α-defensins and immunoglobulin-related genes. These limitations restrict our understanding of the precise mode of action of sSiglec-9. Second, hepatic inflammation and fibrosis were evaluated mainly through histopathology and gene expression, potentially overlooking subtle changes detectable by more sensitive techniques. Third, the sample size in the in vivo experiments was relatively limited, which may constrain the interpretation of statistical robustness. Finally, the use of a single MASH animal model may limit the generalizability of the findings to human disease. Despite these limitations, our results demonstrate the therapeutic potential of sSiglec-9 in modulating gut–liver axis dysfunction and ameliorating hepatic pathology in MASH. These findings provide a foundation for future studies to explore its mechanisms of action and clinical applicability across diverse models and patient populations.

## 5. Conclusions

In this study, we demonstrate that sSiglec-9 significantly restores intestinal barrier function and modulates immune gene expression in the ileum, thereby contributing to the suppression of hepatic inflammation in a MASH mouse model. Although the therapeutic effects of sSiglec-9 on the liver were limited, our data suggest that its primary mode of action is mediated through the gut–liver axis. While further studies are needed to establish causal relationships at the cellular and molecular levels, our findings highlight the therapeutic relevance of targeting intestinal barrier integrity in MASH. sSiglec-9 thus emerges as a promising candidate for future clinical application in MASH and provides a rationale for continued investigation into therapeutic strategies focused on the gut–liver axis.

## Data Availability

The RNA-seq data are available under the accession number PRJNA1225187. Other raw data supporting the conclusions of this study will be made available by the authors upon request.

## Supplementary Materials

The following supporting information can be downloaded at https://www.mdpi.com/article/10.3390/metabo15060366/s1, Table S1: List of differentially expressed genes (DEGs) in the ileum identified by RNA-Seq analysis; Table S2: List of differentially expressed genes (DEGs) in the liver identified by RNA-Seq analysis.

## Abbreviations

The following abbreviations are used in this manuscript:

BW	body weight
ALT	alanine aminotransferase
AST	aspartate aminotransferase
DEG	differentially expressed gene
DMEM	Dulbecco’s Modified Eagle’s Medium
FBG	fasting blood glucose
FITC	fluorescein isothiocyanate
GSEA	gene set enrichment analysis
H&E	hematoxylin–eosin
HGF	hepatocyte growth factor
LEfSe	linear discriminant analysis effect size
MASH	metabolic dysfunction-associated steatohepatitis
MASLD	metabolic dysfunction-associated steatotic liver disease
NAFLD	non-alcoholic fatty liver disease
NAS	NAFLD activity score
PBS	phosphate-buffered saline
SCFA	short-chain fatty acids
SHED	stem cells from human exfoliated deciduous teeth
SHED-CM	stem cells from human exfoliated deciduous teeth-conditioned media
Siglecs	sialic acid-binding immunoglobulin-like lectins
sSiglec-9	soluble Siglec-9

## References

[1] Tateishi R., Uchino K., Fujiwara N., Takehara T., Okanoue T., Seike M., Yoshiji H., Yatsuhashi H., Shimizu M., Torimura T. (2019). A nationwide survey on non-B, non-C hepatocellular carcinoma in Japan: 2011–2015 update. J. Gastroenterol..

[2] Enomoto H., Akuta N., Hikita H., Suda G., Inoue J., Tamaki N., Ito K., Akahane T., Kawaoka T., Morishita A. (2024). Etiological changes of liver cirrhosis and hepatocellular carcinoma-complicated liver cirrhosis in Japan: Updated nationwide survey from 2018 to 2021. Hepatol. Res..

[3] Riazi K., Azhari H., Charette J.H., Underwood F.E., King J.A., Afshar E.E., Swain M.G., Congly S.E., Kaplan G.G., Shaheen A.A. (2022). The prevalence and incidence of NAFLD worldwide: A systematic review and meta-analysis. Lancet Gastroenterol. Hepatol..

[4] Younossi Z.M., Golabi P., Paik J.M., Henry A., Van Dongen C., Henry L. (2023). The global epidemiology of nonalcoholic fatty liver disease (NAFLD) and nonalcoholic steatohepatitis (NASH): A systematic review. Hepatology.

[5] Chan W.K., Chuah K.H., Rajaram R.B., Lim L.L., Ratnasingam J., Vethakkan S.R. (2023). Metabolic dysfunction-associated steatotic liver disease (MASLD): A state-of-the-art review. J. Obes. Metab. Syndr..

[6] Yu J., Shen J., Sun T.T., Zhang X., Wong N. (2013). Obesity, insulin resistance, NASH and hepatocellular carcinoma. Semin. Cancer Biol..

[7] Harrison S.A., Bedossa P., Guy C.D., Schattenberg J.M., Loomba R., Taub R., Labriola D., Moussa S.E., Neff G.W., Rinella M.E. (2024). A phase 3, randomized, controlled trial of resmetirom in NASH with liver fibrosis. N. Engl. J. Med..

[8] Stefan N., Yki-Järvinen H., Neuschwander-Tetri B.A. (2025). Metabolic Dysfunction-Associated Steatotic Metabolic dysfunction-associated steatotic liver disease: Heterogeneous pathomechanisms and effectiveness of metabolism-based treatment. Lancet Diabetes Endocrinol..

[9] Tilg H., Moschen A.R. (2010). Evolution of inflammation in nonalcoholic fatty liver disease: The multiple parallel hits hypothesis. Hepatology.

[10] Marra F., Svegliati-Baroni G. (2018). Lipotoxicity and the gut-liver axis in NASH pathogenesis. J. Hepatol..

[11] Mouries J., Brescia P., Silvestri A., Spadoni I., Sorribas M., Wiest R., Mileti E., Galbiati M., Invernizzi P., Adorini L. (2019). Microbiota-driven gut vascular barrier disruption is a prerequisite for non-alcoholic steatohepatitis development. J. Hepatol..

[12] Muto H., Ito T., Tanaka T., Yokoyama S., Yamamoto K., Imai N., Ishizu Y., Maeda K., Honda T., Ishikawa T. (2021). Conditioned medium from stem cells derived from human exfoliated deciduous teeth ameliorates NASH via the Gut-Liver axis. Sci. Rep..

[13] Matsubara K., Matsushita Y., Sakai K., Kano F., Kondo M., Noda M., Hashimoto N., Imagama S., Ishiguro N., Suzumura A. (2015). Secreted ectodomain of sialic acid-binding Ig-like lectin-9 and monocyte chemoattractant protein-1 promote recovery after rat spinal cord injury by altering macrophage polarity. J. Neurosci..

[14] Ito T., Ishigami M., Matsushita Y., Hirata M., Matsubara K., Ishikawa T., Hibi H., Ueda M., Hirooka Y., Goto H. (2017). Secreted ectodomain of SIGLEC-9 and MCP-1 synergistically improve acute liver failure in rats by altering macrophage polarity. Sci. Rep..

[15] Kang E.A., Soh H., Park S., Lee H.J., Im J.P., Kim J.S. (2020). Soluble Siglec-9 alleviates intestinal inflammation through inhibition of the NF-κB pathway. Int. Immunopharmacol..

[16] Crocker P.R., Paulson J.C., Varki A. (2007). Siglecs and their roles in the immune system. Nat. Rev. Immunol..

[17] Pillai S., Netravali I.A., Cariappa A., Mattoo H. (2012). Siglecs and immune regulation. Annu. Rev. Immunol..

[18] Kukan E.N., Fabiano G.L., Cobb B.A. (2024). Siglecs as modulators of macrophage phenotype and function. Semin. Immunol..

[19] Wang J.H.S., Jiang N., Jain A., Lim J. (2023). Development of effective Siglec-9 antibodies against cancer. Curr. Oncol. Rep..

[20] Wu Y., Huang W., Xie Y., Wang C., Luo N., Chen Y., Wang L., Cheng Z., Gao Z., Liu S. (2022). Siglec-9, a putative immune checkpoint marker for cancer progression across multiple cancer types. Front. Mol. Biosci..

[21] Sakai K., Yamamoto A., Matsubara K., Nakamura S., Naruse M., Yamagata M., Sakamoto K., Tauchi R., Wakao N., Imagama S. (2012). Human dental pulp-derived stem cells promote locomotor recovery after complete transection of the rat spinal cord by multiple neuro-regenerative mechanisms. J. Clin. Investig..

[22] Vacca M., Kamzolas I., Harder L.M., Oakley F., Trautwein C., Hatting M., Ross T., Bernardo B., Oldenburger A., Hjuler S.T. (2024). An unbiased ranking of murine dietary models based on their proximity to human metabolic dysfunction-associated steatotic liver disease (MASLD). Nat. Metab..

[23] Tsuchida T., Lee Y.A., Fujiwara N., Ybanez M., Allen B., Martins S., Fiel M.I., Goossens N., Chou H.-I., Hoshida Y. (2018). A simple diet-and chemical-induced murine NASH model with rapid progression of steatohepatitis, fibrosis and liver cancer. J. Hepatol..

[24] Zhao H., Zhang H., Wu H., Li H., Liu L., Guo J., Li C., Shih D.Q., Zhang X. (2012). Protective role of 1,25(OH)_2_ vitamin D_3_ in the mucosal injury and epithelial barrier disruption in DSS-induced acute colitis in mice. BMC Gastroenterol..

[25] Kleiner D.E., Brunt E.M., Van Natta M., Behling C., Contos M.J., Cummings O.W., Ferrell L.D., Liu Y.C., Torbenson M.S., Unalp-Arida A. (2005). Design and validation of a histological scoring system for nonalcoholic fatty liver disease. Hepatology.

[26] Prieto C., Barrios D. (2020). RaNA-Seq: Interactive RNA-Seq analysis from FASTQ files to functional analysis. Bioinformatics.

[27] Etoh K., Nakao M. (2023). A web-based integrative transcriptome analysis, RNAseqChef, uncovers the cell/tissue type-dependent action of sulforaphane. J. Biol. Chem..

[28] Mu J., Maeda K., Ohashi A., Urano T., Nariai Y., Kamino H., Nakamura M., Yamamura T., Sawada T., Ishikawa E. (2024). Monoclonal antibodies against mature interleukin-18 ameliorate colitis and repair goblet cell function. Dig. Dis. Sci..

[29] Bolyen E., Rideout J.R., Dillon M.R., Bokulich N.A., Abnet C.C., Al-Ghalith G.A., Alexander H., Alm E.J., Arumugam M., Asnicar F. (2019). Reproducible, interactive, scalable and extensible microbiome data science using QIIME 2. Nat. Biotechnol..

[30] Segata N., Izard J., Waldron L., Gevers D., Miropolsky L., Garrett W.S., Huttenhower C. (2011). Metagenomic biomarker discovery and explanation. Genome Biol..

[31] Lu Y., Zhou G., Ewald J., Pang Z., Shiri T., Xia J. (2023). MicrobiomeAnalyst 2.0: Comprehensive statistical, functional and integrative analysis of microbiome data. Nucleic Acids Res..

[32] Ito T., Ishigami M., Zou B., Tanaka T., Takahashi H., Kurosaki M., Maeda M., Thin K.N., Tanaka K., Takahashi Y. (2021). The epidemiology of NAFLD and lean NAFLD in Japan: A meta-analysis with individual and forecasting analysis, 1995–2040. Hepatol. Int..

[33] Tincopa M.A., Anstee Q.M., Loomba R. (2024). New and emerging treatments for metabolic dysfunction-associated steatohepatitis. Cell Metab..

[34] Bergheim I., Moreno-Navarrete J.M. (2024). The relevance of intestinal barrier dysfunction, antimicrobial proteins and bacterial endotoxin in metabolic dysfunction-associated steatotic liver disease. Eur. J. Clin. Invest..

[35] Crouch L.I., Rodrigues C.S., Bakshani C.R., Tavares-Gomes L., Gaifem J., Pinho S.S. (2024). The role of glycans in health and disease: Regulators of the interaction between gut microbiota and host immune system. Semin. Immunol..

[36] Avril T., Floyd H., Lopez F., Vivier E., Crocker P.R. (2004). The membrane-proximal immunoreceptor tyrosine-based inhibitory motif is critical for the inhibitory signaling mediated by Siglecs-7 and-9, CD33-related Siglecs expressed on human monocytes and NK cells. J. Immunol..

[37] Favier B. (2016). Regulation of neutrophil functions through inhibitory receptors: An emerging paradigm in health and disease. Immunol. Rev..

[38] Fu J., Zong X., Jin M., Min J., Wang F., Wang Y. (2023). Mechanisms and regulation of defensins in host defense. Signal Transduct. Target. Ther..

[39] Nakamura S., Nakamura K., Yokoi Y., Shimizu Y., Ohira S., Hagiwara M., Song Z., Gan L., Aizawa T., Hashimoto D. (2023). Decreased Paneth cell α-defensins promote fibrosis in a choline-deficient L-amino acid-defined high-fat diet-induced mouse model of nonalcoholic steatohepatitis via disrupting intestinal microbiota. Sci. Rep..

[40] Simms L.A., Doecke J.D., Walsh M.D., Huang N., Fowler E.V., Radford-Smith G.L. (2008). Reduced α-defensin expression is associated with inflammation and not NOD2 mutation status in ileal Crohn’s disease. Gut.

[41] Mörbe U.M., Jørgensen P.B., Fenton T.M., von Burg N., Riis L.B., Spencer J., Agace W.W. (2021). Human gut-associated lymphoid tissues (GALT); diversity, structure, and function. Mucosal Immunol..

[42] Itoh K., Hirohata S. (1995). The role of IL-10 in human B cell activation, proliferation, and differentiation. J. Immunol..

[43] Paul G., Khare V., Gasche C. (2012). Inflamed gut mucosa: Downstream of interleukin-10. Eur. J. Clin. Invest..

[44] Mukherjee A., Lordan C., Ross R.P., Cotter P.D. (2020). Gut microbes from the phylogenetically diverse genus Eubacterium and their various contributions to gut health. Gut Microbes.

[45] Huang H.S., Lin Y.E., Panyod S., Chen R.A., Lin Y.C., Chai L.M.X., Hsu C.C., Wu W.K., Lu K.H., Huang Y.J. (2023). Anti-depressive-like and cognitive impairment alleviation effects of Gastrodia elata Blume water extract is related to gut microbiome remodeling in ApoE^−^/^−^ mice exposed to unpredictable chronic mild stress. J. Ethnopharmacol..

[46] Chen Y., Tang S. (2023). Gut microbiota and immune mediation: A Mendelian randomization study on granulomatosis with polyangiitis. Front. Immunol..

[47] Cui G., Li S., Ye H., Yang Y., Jia X., Lin M., Chu Y., Feng Y., Wang Z., Shi Z. (2023). Gut microbiome and frailty: Insight from genetic correlation and mendelian randomization. Gut Microbes.

[48] Yamamoto K., Ishigami M., Honda T., Takeyama T., Ito T., Ishizu Y., Kuzuya T., Hayashi K., Goto H., Hirooka Y. (2019). Influence of proton pump inhibitors on microbiota in chronic liver disease patients. Hepatol. Int..

[49] Chen Y., Yang F., Lu H., Wang B., Chen Y., Lei D., Wang Y., Zhu B., Li L. (2011). Characterization of fecal microbial communities in patients with liver cirrhosis. Hepatology.

[50] Qin N., Yang F., Li A., Prifti E., Chen Y., Shao L., Guo J., Le Chatelier E., Yao J., Wu L. (2014). Alterations of the human gut microbiome in liver cirrhosis. Nature.

[51] Hirata M., Ishigami M., Matsushita Y., Ito T., Hattori H., Hibi H., Goto H., Ueda M., Yamamoto A. (2016). Multifaceted therapeutic benefits of factors derived from dental pulp stem cells for mouse liver fibrosis. Stem Cells Transl. Med..

